# Supplementary material for: Authentication of Hippophae rhamnoides ssp. sinensis and ssp. mongolica Based on Single Nucleotide Polymorphism at Ribosomal DNA and Their Vitamin Content Analysis
Source: Plants (Basel). 2022 Jul 14;11(14):1843. doi: 10.3390/plants11141843 (PMC9315697; doi:10.3390/plants11141843)
Supplement: Supplementary file 1 [file plants-11-01843-s001.zip › plants-1702385-supplementary.pdf]

Supplemetry Figure S1. Complete multiple alignment of all SB samples used in this research along with the sequences obtained from NCBI databsae.

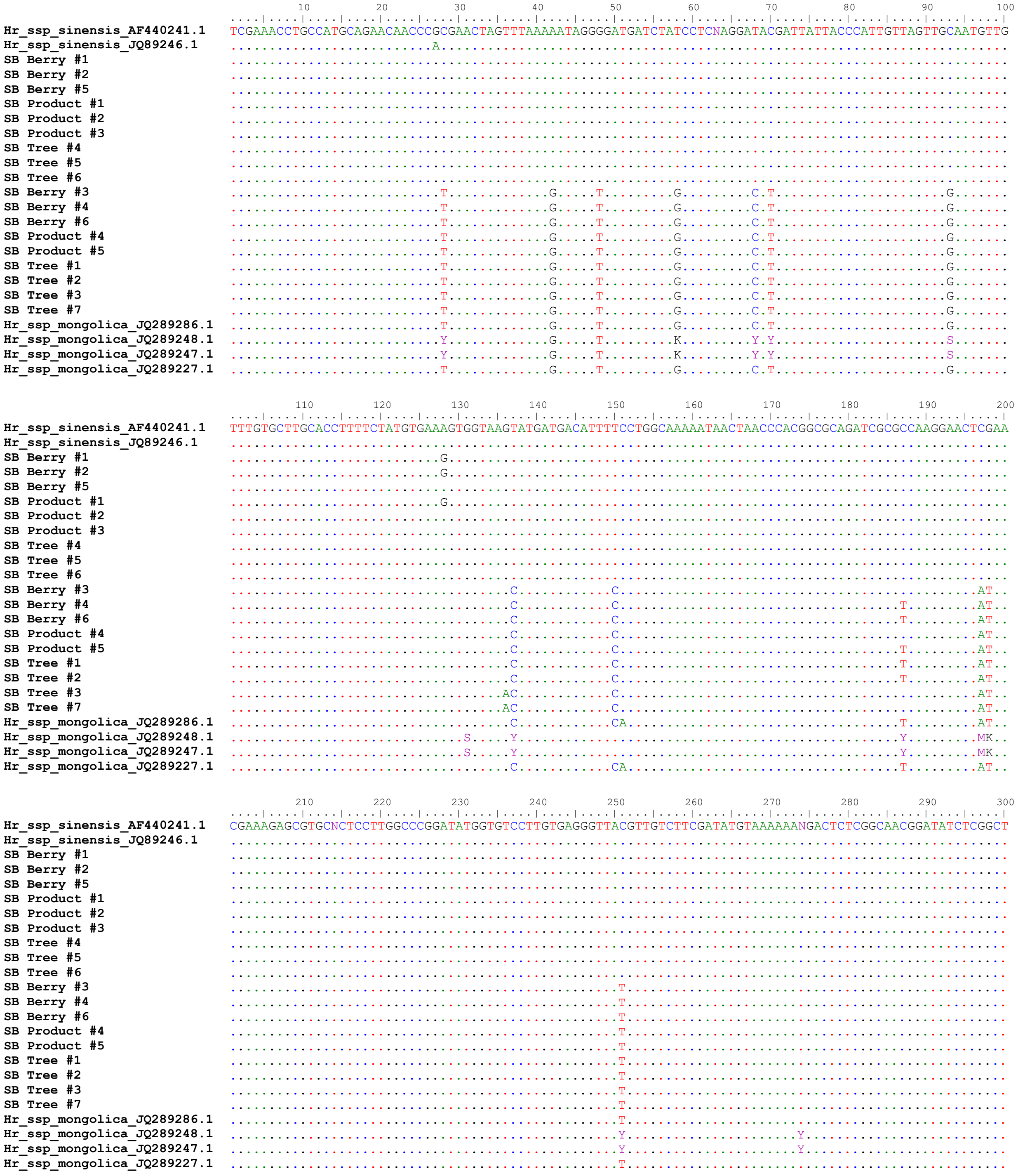

```
                                         310       320       330       340       350       360       370       380       390       400
                                 ....|....|....|....|....|....|....|....|....|....|....|....|....|....|....|....|....|....|....|....|
Hr_ssp_sinensis_AF440241.1       CTCGCATCGATGAAGAACGTAGCGAAATGCGATACTTGGTGTGAATTGCAGAATCCCGTGAACCATCGAGTCTTTGAACGCAAGTTGCGCCCGAAGCCAT
Hr_ssp_sinensis_JQ89246.1        ....................................................................................................
SB Berry #1                      ....................................................................................................
SB Berry #2                      ....................................................................................................
SB Berry #5                      ....................................................................................................
SB Product #1                    ....................................................................................................
SB Product #2                    ..T.................................................................................................
SB Product #3                    ....................................................................................................
SB Tree #4                       ....................................................................................................
SB Tree #5                       ....................................................................................................
SB Tree #6                       ....................................................................................................
SB Berry #3                      .............................T..............................................................A.......
SB Berry #4                      .............................T..............................................................A.......
SB Berry #6                      .............................T..............................................................A.......
SB Product #4                    .............................T..............................................................A.......
SB Product #5                    .............................T..............................................................A.......
SB Tree #1                       .............................T..............................................................A.......
SB Tree #2                       .............................T..............................................................A.......
SB Tree #3                       .............................T..............................................................A.......
SB Tree #7                       .............................T..............................................................A.......
Hr_ssp_mongolica_JQ289286.1      .............................T......................................................................
Hr_ssp_mongolica_JQ289248.1      .............................Y..............................................................R.......
Hr_ssp_mongolica_JQ289247.1      .............................Y..............................................................R.......
Hr_ssp_mongolica_JQ289227.1      .............................T..............................................................A.......

                                         410       420       430       440       450       460       470       480       490       500
                                 ....|....|....|....|....|....|....|....|....|....|....|....|....|....|....|....|....|....|....|....|
Hr_ssp_sinensis_AF440241.1       CAGGCTGAGGGCACGTCTGCCTGGGCGTCACACACCGTTGCCCTCCCAACATCACGTTCCTTCAAGGCTACGTTGGTTGTGAAGGCGCATATTGGCTTCC
Hr_ssp_sinensis_JQ89246.1        ....................................................................................................
SB Berry #1                      ....................................................................................................
SB Berry #2                      ....................................................................................................
SB Berry #5                      ....................................................................................................
SB Product #1                    ....................................................................................................
SB Product #2                    ....................................................................................................
SB Product #3                    ....................................................................................................
SB Tree #4                       ....................................................................................................
SB Tree #5                       ....................................................................................................
SB Tree #6                       ....................................................................................................
SB Berry #3                      ....................................................................................................
SB Berry #4                      ....................................................................................................
SB Berry #6                      ....................................................................................................
SB Product #4                    ....................................................................................................
SB Product #5                    ....................................................................................................
SB Tree #1                       ....................................................................................................
SB Tree #2                       ....................................................................................................
SB Tree #3                       ....................................................................................................
SB Tree #7                       ....................................................................................................
Hr_ssp_mongolica_JQ289286.1      ....................................................................................................
Hr_ssp_mongolica_JQ289248.1      ....................................................................................................
Hr_ssp_mongolica_JQ289247.1      ...............................................................................................Y....
Hr_ssp_mongolica_JQ289227.1      ....................................................................................................
```

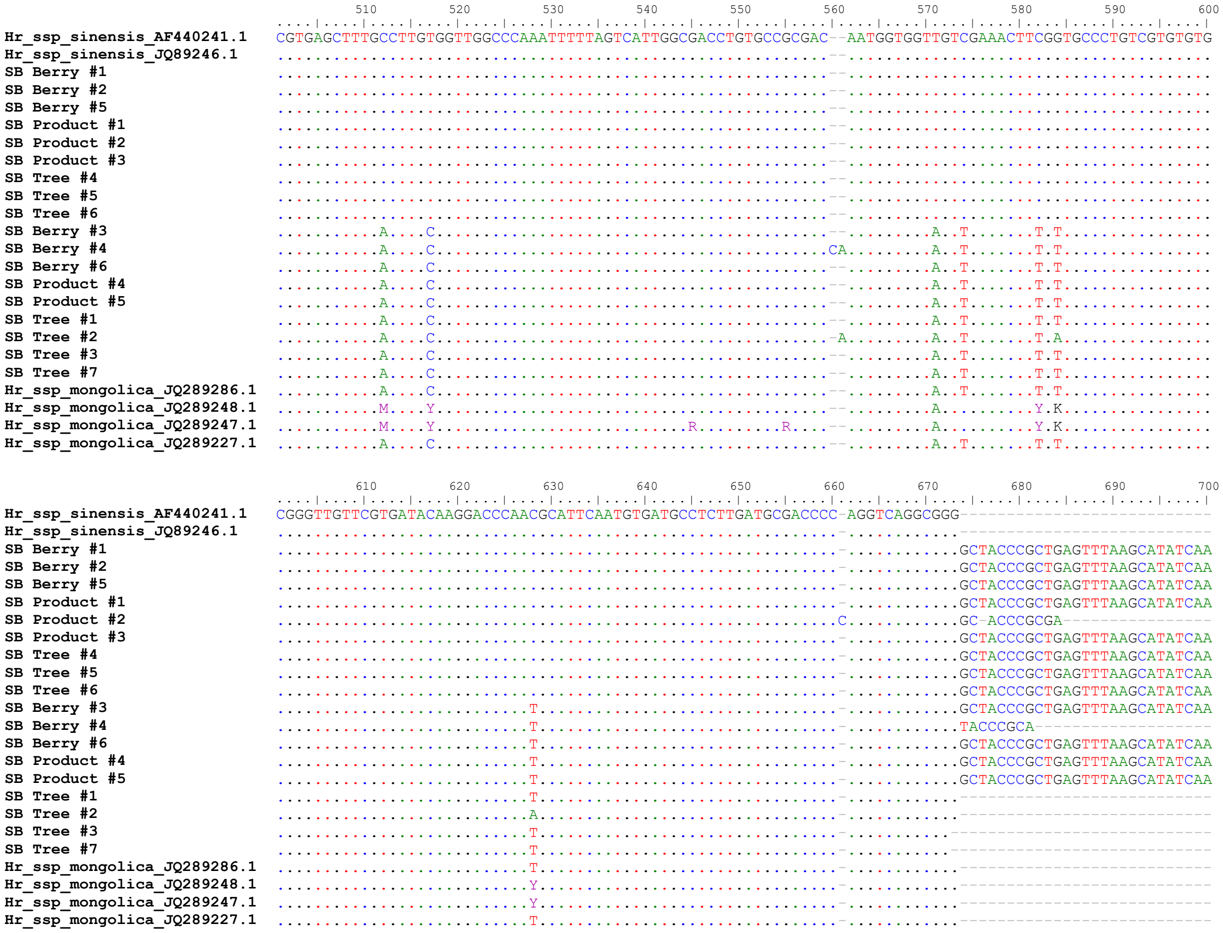
510 520 530 540 550 560 570 580 590 600
Hr_ssp_sinensis_AF440241.1
Hr_ssp_sinensis_JQ89246.1
SB Berry #1
SB Berry #2
SB Berry #5
SB Product #1
SB Product #2
SB Product #3
SB Tree #4
SB Tree #5
SB Tree #6
SB Berry #3
SB Berry #4
SB Berry #6
SB Product #4
SB Product #5
SB Tree #1
SB Tree #2
SB Tree #3
SB Tree #7
Hr_ssp_mongolica_JQ289286.1
Hr_ssp_mongolica_JQ289248.1
Hr_ssp_mongolica_JQ289247.1
Hr_ssp_mongolica_JQ289227.1
610 620 630 640 650 660 670 680 690 700
